# Interaction microanalysis of foster care research using THEME

**DOI:** 10.3389/fpsyg.2022.956259

**Published:** 2022-10-18

**Authors:** Pauline Simon, Alain Blanchet

**Affiliations:** Vulnerability, Capability, Rehabilitation Team (VCR), Ecole de Psychologues Praticiens of the Institut Catholique de Paris, Paris, France

**Keywords:** interaction analysis, foster care, emotion regulation, clinical psychology, pattern detection

## Abstract

Environmental stress is a key element to the understanding of the psychopathology of children in foster care. Such children often present a wide range of symptoms from anxiety to depression, including abnormal behaviors in their interactions with adults that can be related to experience suffered in their family of origin (e.g., abandonment, abuse, etc.). Foster care should provide a safe environment, both to protect children from abuse and to help them build a well-adjusted developmental trajectory. The relationships with the family of origin may also be maintained. How do children in foster care behave in relation to caregivers given the differences between the families they grow up in? This study focuses on three adult-child relationships: those with a foster carer, a mother and a father. Each adult-child interaction was recorded several times in a day-to-day environment. On each occasion the instruction was given to behave naturally while interacting with a child. No additional material was supplied. Our observations concern the verbal and non-verbal comportment of a 4-year-old foster child named Julia when entering the study, with her caregivers. Once the principal elements had been coded (behaviors, verbalizations), a sequential behavioral patterns analysis was performed using the THEME© program. For this purpose, a 2-min interaction was chosen from the third video of an event which appeared particularly representative of the relationship between Julia and her different caregivers. According to whom Julia was with, the results reveal very different interactive processes. We observe, for example, that with the foster carer the interaction patterns were primarily focused on play objects, whereas they involved more collaborative activity with the father and distraction/avoidance behaviors with the mother. The study identifies the use of disengaging and self-exciting behaviors in all types of interaction. Those emotion regulation strategies are particularly developed during parent–child sessions, showing pathological processes of relationship.

## Introduction

In France, more than 20,000 children under 6 years old are in foster care; this represents 4.5‰ of the child population in that age range and 14.3% of the children outplaced for protection [Observatoire National de la Protection de l’Enfance (ONPE), 2019]. This number has been stable over the past 15 years, the psycho-emotional developmental conditions among foster children needs attention. Serious neglect, psychological or physical violence within a family are the main reasons for placement. Such dangers faced by children oblige a judge to pronounce parent–child separation if health, safety or morality are deemed to be seriously compromised (Civil Code, 2016). Foster care is intended to provide protection for a child while maintaining his or her bonds with the biological parents. In France, the primary objective is always to restore family life so that the child can return to his or her original home.

Abused children face experiences that severely compromise their emotional, cognitive, and social development (Cicchetti et al., 2016). All of these damaging experiences need to be considered in relation to a child’s maturation and developmental age in order to understand how adverse experience impacts on emotion regulation development.

### Negative consequences of abuse and placement

The proportion of prolonged and repeated experiences of trauma (Villodas et al., 2016) among foster children is high (Vasileva and Petermann, 2017). This has a considerable impact on the child’s attachment style, which is often insecure or disorganized (Van Ijzendoorn et al., 1999). Attachment theory provide a framework to understand how interactive processes lead to the internalization of attachment through Internal Working Models (Bowlby, 1980). Attachment is considered to be secure and insecure-anxious/avoidant, insecure-anxious/ambivalent or insecure-disorganized. Disorganized behaviors are associated with a constant need for reassurance and comfort, while impulsive attitudes and a lack of inhibitory control may develop in a child’s relationships with caregivers and peers leading to severe mental illness processes (Kim and Cicchetti, 2010). The authors were evaluating the links between heterogenous experience of maltreatment and emotion dysregulation and psychopathology. They found that cumulative risks of maltreatment are related to emotion dysregulation, internalized and externalized symptoms and negative peer relationships. The more a child has experienced traumatic events, the more likely he or she is to present high levels of disorganization and symptoms of Post-Traumatic Stress Disorder and depression (Haselgruber et al., 2021).

The role of the foster carer is to provide a stable and secure setting for abused children (Dozier, 2005; Yarger et al., 2020). To this end, it is essential to work to reduce stress in the child’s environment (Healey and Fisher, 2011; Vasileva and Petermann, 2017). The capacity of caregivers to understand and adapt to a child’s needs is crucial to developing resilience and reducing symptoms (Dubois-Comtois et al., 2015; Yarger et al., 2020). Professional training based on developing mentalization processes and sensitivity, help to enhance the foster child’s safety, well-being and needs satisfaction (Fonagy et al., 2004; Steenbakkers et al., 2018).

### Impact on emotion regulation development

Emotion Regulation (ER) refers to the ability to be aware of one’s emotions to the extent that it is possible to modify their intensity and temporality toward the achievement of a specific goal (Thompson, 1994). ER develops in the interactions that very young children have with their environment through a bio-feedback process (Gergely and Watson, 1999). This mechanism consists of mirroring a child’s emotions so that he/she is made aware of what is expressed and so encouraged to recognize it. By learning Emotion Regulation Strategies (ERS) in interpersonal relationships, children develop and consolidate future internalized ERS (Holodynski and Friedlmeier, 2011; Thompson, 2014), the whole process taking place within the family (Morris et al., 2007). When encountering a new attachment figure, however, such as a foster carer who may have different parenting practices from those of a child’s natural parents, many children are potentially disturbed. Emotional self-regulation, arising in infancy as a child’s response to the reactions of those around him or her, reflects a child’s need to find an appropriate balance between security and stimulation (Cole and Deater-Deckard, 2009).

It is for this reason that it is important to focus on disorganized behaviors and ERS when working with foster children (Haselgruber et al., 2021), since a foster carer’s generally greater sensitivity to a child’s difficulties tends to reduce externalizing behaviors and improve both emotional security and cognitive and socio-emotional development (Poitras et al., 2021). It is particularly in language strategies – promoting verbal communication and cognitive reappraisal – that foster carer-child interactions provide support to a child’s development and resilience (Simon-Herrera et al., 2022). Although foster care has as a primary objective to sustain child development and restore family life, there is moderate evidence that parent–child visits are often related to higher likelihood of externalizing symptoms manifested by a lack of behavioral control and psychomotor instability (Poitras et al., 2021). Thus, while foster care may be beneficial, there remains a risk that contact between parent and child during placement will tend to sustain or reinvigorate the impact of previous traumatic experiences.

### Objective of the study

The aim of this study is to analyze the ERS processes in child-caregiver relationships. Children evolve in an environment where, in educational, affective and emotional terms, they may have several parental references. The observational methodology and microanalysis used here is exclusively concerned with the ER processes of one particular child in foster care. The study aims at giving a case-report of a foster child interplay with caregivers and to support a fruitful use of ethological methods such as video-based analysis of behaviors and T-pattern analysis.

It was expected that the interactions between child and foster carer would show a higher rate of verbal exchange and mental elaboration, whereas in those between child and parents, a greater negativity and lack of emotional control were expected. Because foster carer are trained professionals, their level of positivity would ensure warmer interactions processes supported by adult-child conversations.

## Materials and methods

An exploratory, clinical study to analyze ER learning processes was conducted within the French child protection service. To illustrate that study, a single case is detailed here: that of Julia, a foster child aged 4. She was chosen because she showed particularly clearly how a foster child can develop heterogeneous ER processes that may range widely from suitably adapted to highly pathological mechanisms. Julia showed a wide variability in her behavior according to whom she was interacting with: mother, father or foster carer. Her attitudes were primarily evaluated by the psychologist proceeding at the video-recording through direct expert observation. She was observed through video-recording with her caregivers: mother, father and foster carer. Each session lasted 1 h and were replicated three times with every caregiver for a total of nine sessions. Meetings between Julia and her parents occurred separately respecting the parent–child conditions of visits authorized by the judge. The video material was then transcripted and coded according to ERS observed.

The use of THEME© software allowed us to study in detail how ERS manifested in the course of interactions.

### Participant recruitment and data collection

Adult participants were contacted by telephone and were offered the opportunity to participate in a study assessing child development in foster care, based on interactions between child, parents and foster carer. During this phone call, it was explained that the meetings would take place in the child’s usual home circumstances and that they would be videotaped to allow further detailed analysis. Observations were made within the framework set out for parent–child visits and at the foster carer’s home. Julia resided with her foster carer for 19 months. Julia and her caregivers knew the psychologist proceeding at the video-recording since the beginning of her placement because of the clinical work done when entering and following the development of Julia in foster care. The actual recordings took place at home, in the living room, with the foster carer and in a supervised visits room for each parent. In France, foster care is a controlled and regulated profession whose purpose is to provide a secure home for foster children. The French welfare system does not consider the foster carer as a parent but as a professional only. For this reason, the carer’s own family did not participate in the study.

A consent form was signed by all participants, parents signing for their child. This form included information about both the design of the study and the video recordings. It also included information about: the confidentiality of the data, the anonymity of the participants, the possibility of withdrawing participation at any time or refusing to answer (without any consequences for the person), the possibility of obtaining additional information and the fact that the research would result in scientific publication.

Data, drawn from the video-recordings, were collected on three occasions at six-monthly intervals. Observation in the child’s usual environment allowed for a closer look at the child’s reality and to perceive the real conditions of adult-child interactions. The psychologist, who was present throughout the recordings, asked the participants to behave normally and offered no indications as to any expectations. No specific material was given. A debriefing time to collect the adult’s feelings about the session and to discuss the child’s behaviors, was systematically held at the end of each recording. The primary function of this period was to verify that the recorded scenes corresponded to the adults’ perception of their own and their children’s usual behavior, since it was understood that the presence of a video camera could have an impact on spontaneity. From an ethical point of view, any problematic adult behavior was discussed with the person concerned in order to find the best strategy to help the child.

#### Presentation of Julia

Julia was a 4-year-old girl, placed in foster care at the age of 2 years 5 months, worryingly thin and in a state of severe undernutrition for which there was no known organic cause. This anorexia was accompanied by a major delay in development and behavioral problems (rocking). Julia’s parents both had addictions: her father, who had suffered physical abuse in his own family as a child, to alcohol; her mother, whose father had abandoned her, to cannabis. Julia was taken in by a foster carer who described her as having food refusal behaviors, vomiting and significant psychomotor instability. Unable to control her psychomotor impulses, Julia’s behavior was reported to be massively disorganized even at school. Julia could also exhibit compulsive masturbation. She received psychological, medical and speech therapy help. She always met with her parents in a neutral place and saw them separately every 2 weeks. Under the responsibility of the child protection service, she had one visit per week with one of her parents.

### Analysis of the data

Child’s and adults’ behaviors were coded, to assess the ERS used in their interactions. Double coding was performed and led to significant consistency between the two. An iterative method was preferred for its degree of relevance to the calculation of the degree of concordance between observers not involved in the study problem.

The coding system used in this study was analyzed regarding behaviors that can be related to ERS observed in adults and children. From the videographic records, sequences were first transcripted in details and then behaviors were coded following the ERS that are presented below. The Table 1 give the details of all coded behaviors.

**Table 1:** Coded ERS and behaviors.

Coded ERS child	Coded ERS adults	Coded ERS all participants	Coded behaviors all participants	Coded basic emotions all participants
Engagement object	Comfort	Help seeking: examiner, pe (father), me (mother), fc (foster carer)	Calm voice	Joy
Disengagement	Attention refocusing	Cognitive reevaluation	Cheerful voice	Anger
Self-soothing	Instrumental strategies		Groan	Fear
Self-excitement	Emotion expression positive or negative		Smile	Sadness
Physical venting	Identify the child’s emotions		Laugh	Disgust
Escape			Agressive voice	
Visual exploration			Ferm voice	
Positive and negative verbalization			Sigh	

*The child’s ERS* were assessed on each of the recordings in terms of adult-child interactions. Strategies used by the child were coded according to the following behaviors: *Engagement object* (concerning a child’s ability to focus and be attentive to things or tasks presented); *Disengagement* (seeking out or manipulating an object different from the one involved in a current action); *Self-soothing* (self-manipulative behavior, e.g., sucking a thumb or touching the hair); *Self-excitement* (e.g., clapping hands, singing, or talking to self); *Physical venting* (e.g., throwing an object, hitting, banging in play); *Escape* (e.g., fleeing from an uncomfortable situation as it develops); *Visual exploration* (looking for visual distractions without completely turning away from an activity in progress) selected from the studies of Blandon et al. (2010) and *positive or negative verbalization* (the verbal expression of emotion in speech, e.g., yelling or giggling).

*Adults’ ERS* were assessed in the same way. The strategies used by the adults were coded according to the following behaviors: *Comfort* (physical or verbal behaviors, e.g., hugging); *Attention Refocusing* (attempts to redirect a child’s – or the adult’s own – attention to another stimulus); and *Instrumental strategies* (changing a situation or eliminating a source of frustration) selected from the work of Morris et al. (2011). Other ERS can be added: *Emotion expression positive or negative* (e.g., expressing joy or showing aggression). Finally, an important parenting skill is the ability to *identify a child’s emotions:* this ERS is a form of cognitive reappraisal (Holodynski and Friedlmeier, 2011).

Some strategies were coded for adult and child: *Help seeking* (a willingness to be with a person or to vocalize for help for children/attempting to get help with emotional regulation from a third party) and *Cognitive reevaluation* (reinterpreting and explaining a situation to an adult/a child) from Mikolajczak et al. (2016).

In addition to the ERS that had been previously coded, behaviors were specified to further detail the correspondence between ERS and body, expressive, or tonal attitude. For example, tones of voice were specified based on whether the tone was perceived as calm, firm or even cheerful, to characterize – as positive or negative – the type of verbalization/expression. Laughter attitudes were also reported. Finally, the perceived basic emotions – joy, anger, fear, sadness, disgust, (Ekman, 2016) – were associated with positive or negative expression. We give some examples of how emotional behaviors and basic emotions are coded: laughing is coded as a visual and auditive manifestation; smiling is coded as a visual manifestation only; joy is expressed inside the interaction, with multiple body gestures and is related to the emotional context of the relationship. Joy implies behaviors that are more complex that smiling and laughing only.

To have an in-depth exploration of the interactive processes in adult-child relationship, THEME6EDU© software was chosen because it is able to detect interaction patterns invisible to the naked eye: the algorithm it employs recognizes T-patterns, that is, patterns that emerge from the analysis of behaviors/events over time and level by level and, in our case, specifies that the fractals should include feedback loops characterized by symmetric translation (Magnusson, 2017). The T-Pattern analysis “has allowed the description and detection of intra- and inter-individual causal and non-causal patterns frequently sharing the T-pattern structure, but the detection of intra-individual patterns may be a precondition for the detection of more complex inter-individual patterns” (Magnusson, 2020). For this microanalysis a 2-min interaction deemed particularly representative of the adult-child relationship, was selected from all the recordings of the child’s interactions with caregivers; a similar sequence occurred repeatedly with a significance in the THEME© software where *p* < 0.005. The choice of sequence was also guided by an analysis of clinical elements. Video analysis was performed in order to identify interactional patterns that were replicating within relationships. The adult reacts to the child’s ERS and with feedback the child will respond to this adult input and will adjust his or her own ERS. The video micro-analyses provided essential information on the deep structure of interactions and allowed us to identify a network of interacting loops (Simon-Herrera and Duriez, 2021). Only the most complex and heuristic patterns are presented. The THEME© software also provides information about occurrences of behaviors and ERS as well as percent values of interaction initiated by Julia with her different caregivers. To ensure the validity of the data, the Monte Carlo test was performed on THEME©. This test consists of comparing the number and complexity of the real data versus the randomized data to obtain statistical validation.

Debriefing and video-feedback with the participants ensured that the coding matched the emotional intent expressed by the adults. The child’s behaviors were debriefed with the adult only.

## Results

### Occurrence of behaviors by type of interaction

Table 2 shows the occurrence of ERS by adult and by child in the different interactions. Those occurences are directly generated by the THEME software and give a first look at the most used ERS in each type of interaction.

**Table 2:** Occurrences of behaviors for each interaction.

Julia and her FC	Julia and her mother	Julia and her father
1 ju, engagementobject (41)	1 ju, selfexcitement (43)	1 pe, cognitivereevaluation (34)
2 fc, calmvoice (37)	2 ju, positiveverbalization (33)	2 pe, calmvoice (33)
3 fc, cognitivereevaluation (33)	3 ju, engagementobject5 (29)	3 ju, positiveverbalization (29)
4 ju, positiveverbalization (30)	4 ju, disengagement (17)	4 ju, selfexcitement (25)
5 ju, cognitivereevaluation (24)	5 ju, engagementobject2 (17)	5 ju, cognitivereevaluation (20)
6 ju, calmvoice (17)	6 ju, joy (16)	6 ju, calmvoice (17)
7 ju, selfexcitment (14)	7 ju, engagementobject (15)	7 ju, helpseekingexaminer (12)
8 ju, visualexploration (12)	8 ju, cognitivereevaluation (13)	8 ju, joy (12)
9 ju, helpseekingfc (11)	9 ju, calmvoice (12)	9 pe, instrumentalstrategy (11)
10 ju, cheerfulvoice (10)	10 ju, helpseekingexaminer (12)	10 ju, engagementobject2 (10)
11 ju, helpseekingexaminer (10)	11 ju, cheerfulvoice (10)	11 ju, laugh (10)
12 fc, instrumentalstrategy (9)	12 ju, laugh (10)	12 ju, disengagement (6)
13 ju, selfsoothing (9)	13 me, smile (10)	13 pe, smile (6)
14 fc, helpseekingexaminer (8)	14 ju, visualexploration (8)	14 ju, agressivevoice (5)
15 ju, joy (8)	15 ju, helpseekingme	15 ju, cheerfulvoice (5)
16 fc, attentionrefocusing (7)	16 ju, smile (7)	16 ju, negativeverbalization (5)
17 ju, negativeverbalization (5)	17 me, calmvoice (6)	17 ju, engagementobject3 (4)
18 ju, disengagement (4)	18 me, cognitivereevaluation (5)	18 ju, physicalventing (4)
19 ju, groan (4)	19 ju, engagementobject3 (4)	19 ju, anger (3)
20 ju, physicalventing (4)	20 ju, physicalventing (4)	20 ju, engagementobject (3)
21 ju, smile (4)	21 me, helpseekingexaminer (4)	21 ju, helpseekingpe (3)
22 ju, laugh (3)	22 ju, engagementobject4 (2)	22 ju, smile (3)
23 ex, cognitivereevaluation (2)	23 me, attentionrefocusing (2)	23 ju, groan (2)
24 fc, emotionexpressionnegative (2)	24 me, instrumentalstrategy (2)	24 ju, visualexploration (2)
25 ju, fear (2)	25 ex, cognitivereevaluation (1)	25 pe, attentionrefocusing (2)
26 fc, agressivevoice (1)	26 ju, engagementobject6 (1)	26 pe, helpseekingexaminer (2)
27 fc, cheerfulvoice (1)	27 ju, groan (1)	27 pe, cheerfulvoice (1)
28 fc, comfort (1)	28 me, cheerfulvoice (1)	28 pe, emotionexpressionnegative (1)
29 fc, fermvoice (1)		
30 fc, smile (1)		
31 ju, agressivevoice (1)		
32 ju, sad (1)		
33 ju, sigh (1)		

Ju: Julia; fc, foster carer; me, mother; pe, father. Helpseeking is followed by fc, me, pe or examiner regarding the person that is called for support. Engagementobject is often followed by a number which indicates the number of objects Julia is playing with and how long she actually plays with every object (e.g., with her foster carer she only played with one object with 41 occurrences while she invested six objects with her mother).

**Figure 1:**
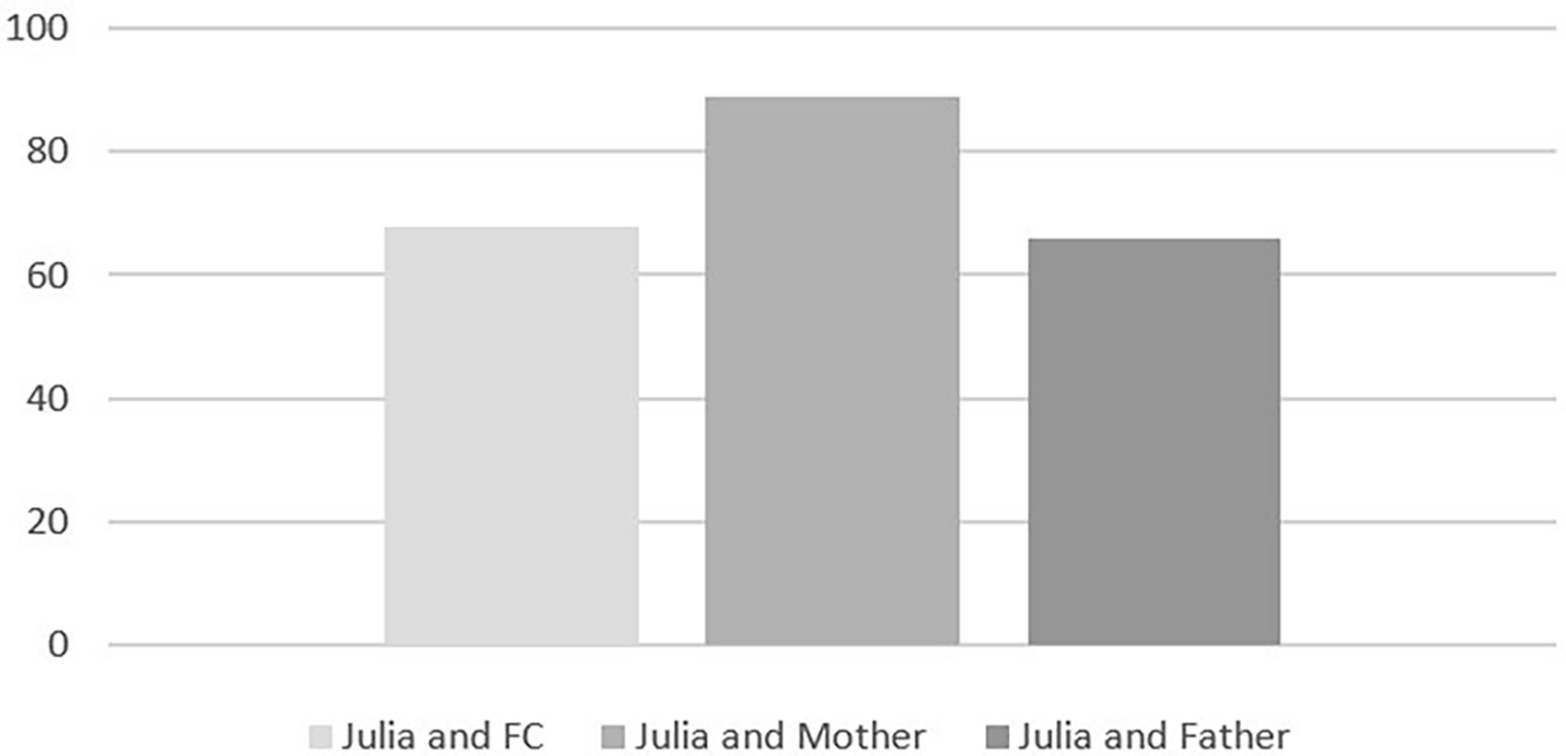
Interaction initiated by Julia in %.

Figure 1 shows how often Julia initiated the interaction with her caregivers.

We can see that the percentage of interaction initiation of Julia is superior with her mother rather than when she is with, her foster mother or her father.

It indicates that 68% of the interactions between Julia and her foster carer were initiated by Julia. Her behaviors were focused on engagement with an object while the foster carer spoke with her in a calm voice, employing cognitive reassessment; Julia similarly showed a preference for positive verbalizations and language elaboration.

In the company of her mother, 89% of acts were performed by Julia. Self-excitement behaviors (e.g., making noises, jumping up and down) were most present in this relationship. There were both numerous avoidance behaviors and multiple object engagements. The table also shows that Julia’s mother was very passive in the interaction, mostly smiling for instance.

In the presence of her father, 66% of actions were performed by Julia. In a calm voice he would frequently use cognitive reappraisal; she would tend to use the same strategies while exhibiting self-exciting attitudes. Her engagements with objects were less frequent while he tended to use more instrumental strategies. In this interaction Julia was more prone to seek support from the psychologist present.

The overall tone of the interactions remained positive in each case.

### Interaction patterns analysis

Analysis of interaction patterns, derived from analyses performed with the THEME© software, highlights the processes and behavioral chains that are established during exchanges between the child and each of her reference adults.

**Figure 2:**
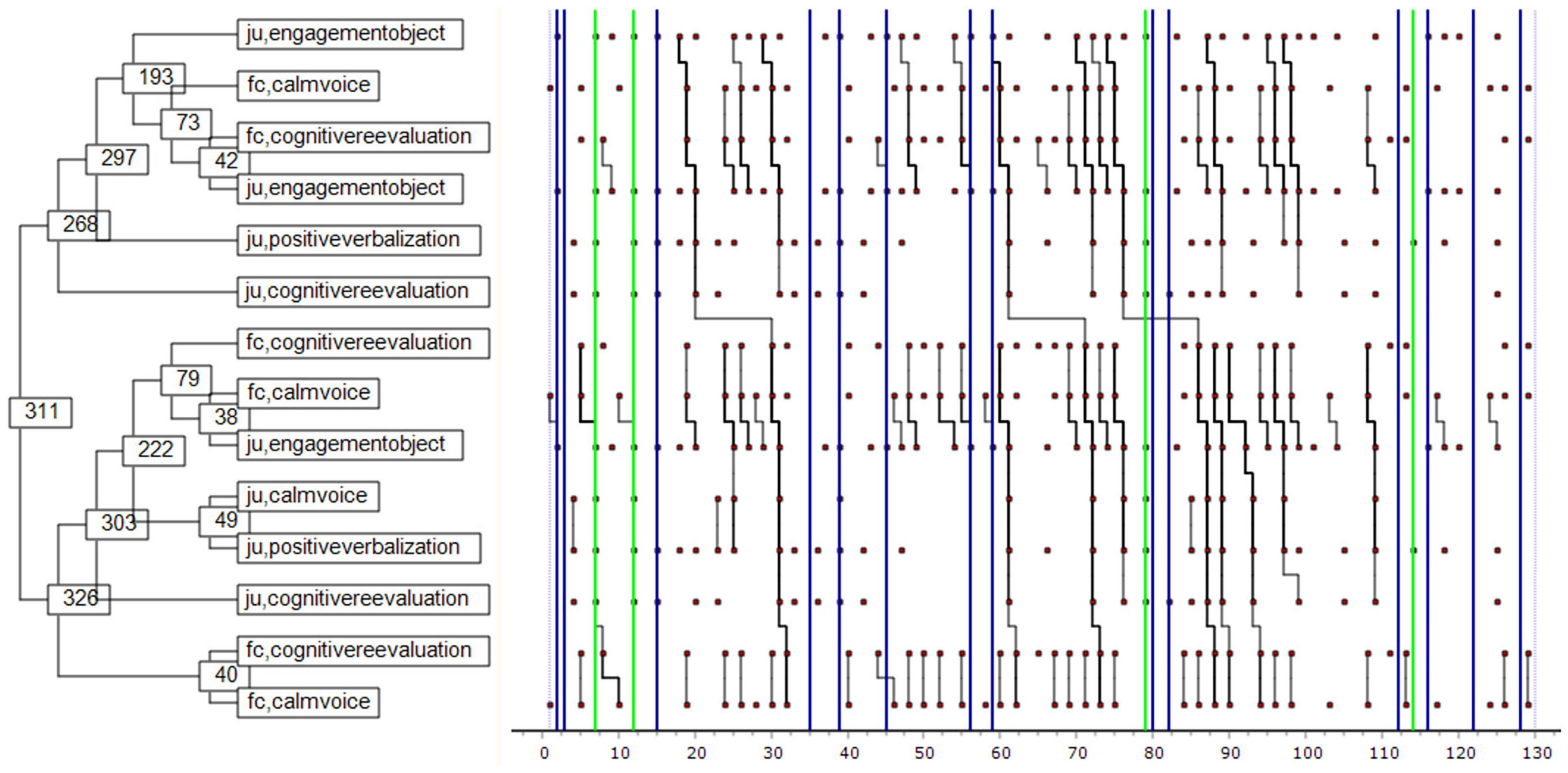
Julia-Foster Carer interaction pattern. ju: Julia, fc: foster carer. The X-axis shows the number of behavioral units occurring within the selected 2-min interaction (129 acts overall). Green lines highlight the disengagement behaviors and blue lines are related to self-excitement attitudes. The numbers appearing in the left side of the figure are related to an identification number of each node and is not related with the appearing occurrence of each behavior.

**Figure 3:**
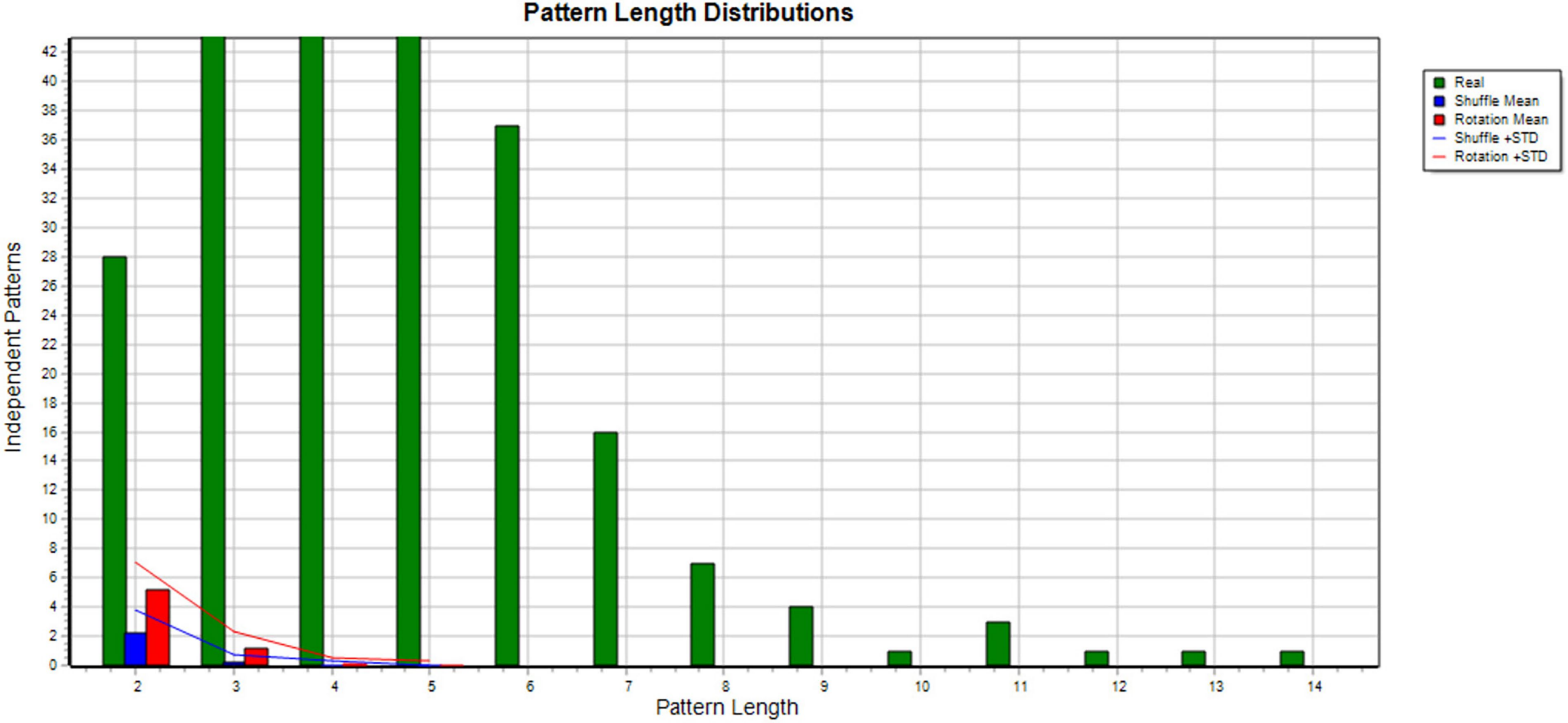
Monte Carlo test results. Shows the results of T-shuffling (blue) and T-rotation (red) for TPA of Julia and her foster carer interactions where 282 patterns were found. The difference between real and randomized data is important and no are found in the randomized data with the longer patterns.

Figure 2 shows the patterns of interaction between Julia and her foster carer. Together they performed 129 acts. It shows that when Julia was engaged with an object (e.g., painting, playing with a doll), her foster carer spoke in a calm voice, prompting cognitive reappraisal and reinforcing Julia’s object engagement. This in turn led to positive verbalization and mental elaboration by both Julia and her foster carer, who continued the exchange in a conversational mode so long as Julia remained engaged with an object. The blue lines indicate the self-excitation behaviors that occurred, mainly during pauses in this pattern. The green lines indicate the rare instances of disengagement when distraction behavior was employed. The pattern of interaction in general between Julia and her foster carer indicates a tendency toward thoughtful action and collaboration. The Monte Carlo results are shown in Figure 3.

**Figure 4:**
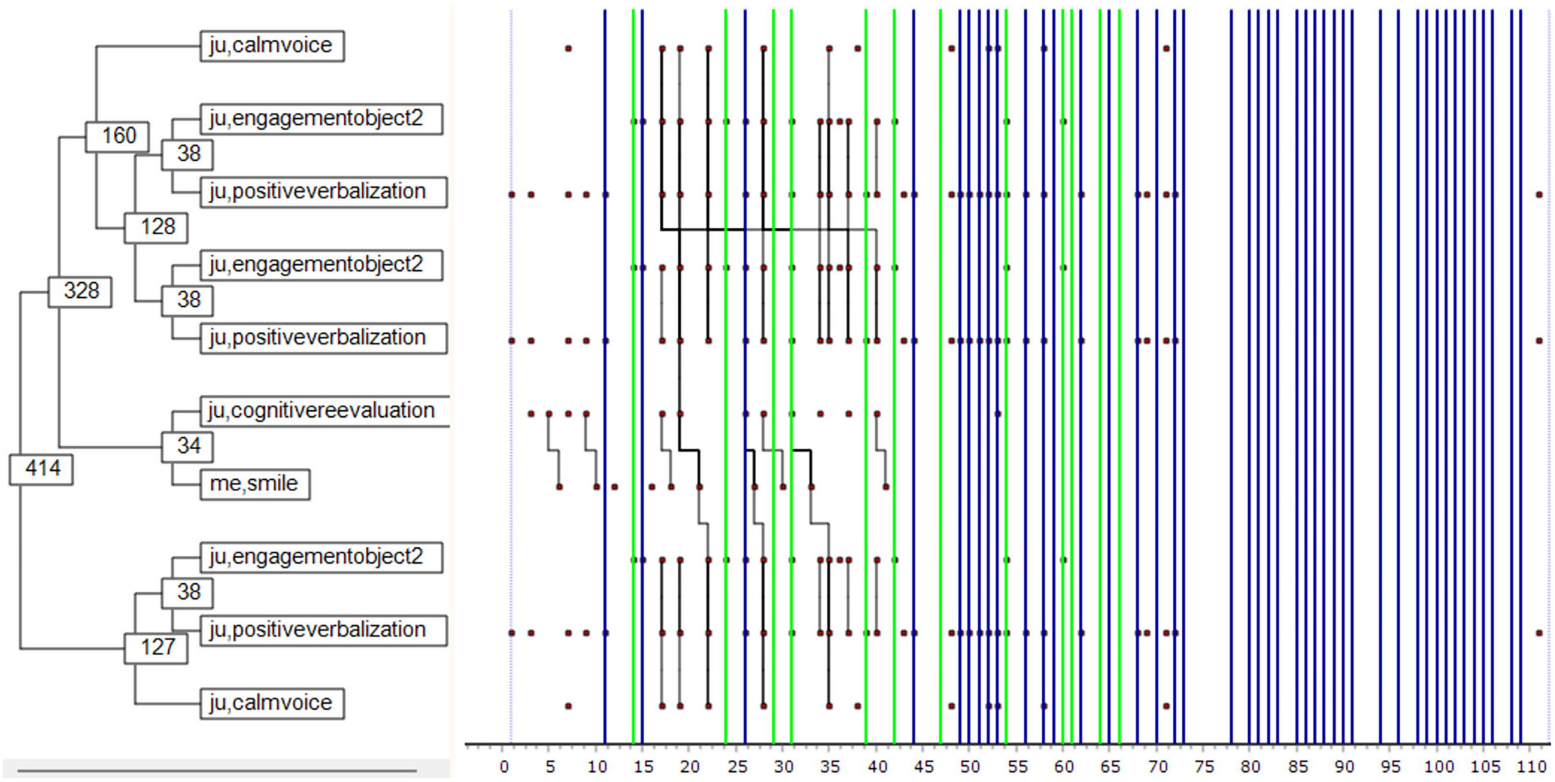
Julia-Mother interaction pattern. ju: Julia, me: mother. The X-axis shows the number of behavioral units occurring within the selected 2-min interaction (111 acts overall). Green lines highlight the disengagement behaviors and blue lines are related to self-excitement attitudes. The numbers appearing in the left side of the figure are related to an identification number of each node and is not related with the appearing occurrence of each behavior.

**Figure 5:**
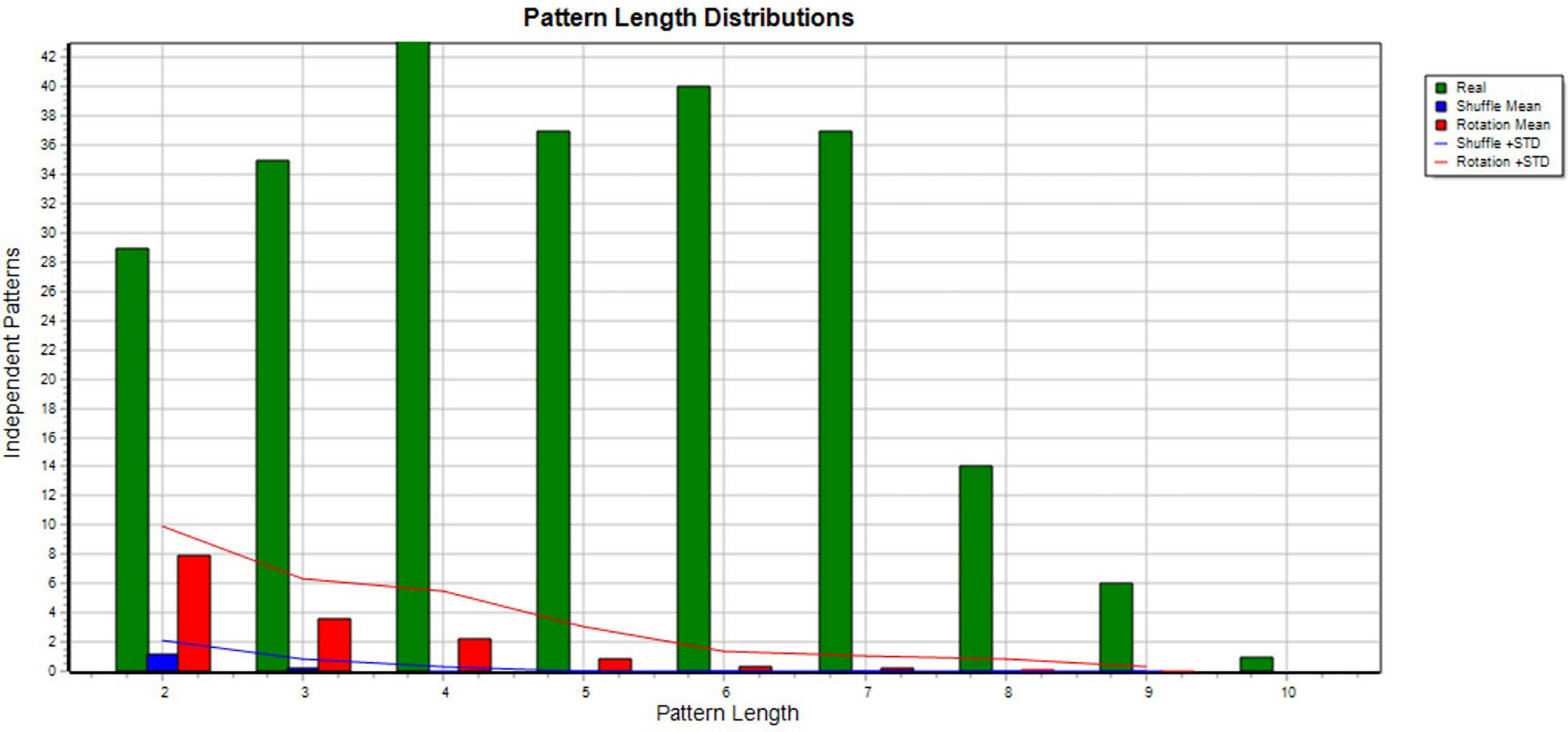
Monte Carlo test results. Shows the results of T-shuffling (blue) and T-rotation (red) for TPA of Julia and her mother interactions where 293 patterns were found. The difference between the real and randomized data is great and with longer patterns, none are found in the randomized data.

Figure 4 shows the patterns of interaction between Julia and her mother. Together they performed 111 acts. It shows that when Julia interacted with her mother and spoke calmly, she tended to be engaged with an object and would produce positive verbalizations. This sequence recurred and led Julia to cognitive reappraisal, triggering a maternal smile. Julia then re-engaged with the object, verbalizing positively. In this episode Julia was more or less the only active participant; her mother reacted little and largely non-verbally. Disengagement is significant in the first half of the sequence (green lines) and is followed by a succession of self-excitation events (blue lines) where the interactive pattern no longer appears in the background. The relationship between Julia and her mother here suggests that the child is acting alone. Object engagement is really more a question of object manipulation (e.g., dolls, cubes, boxes) than any deliberately developed game. The Monte Carlo results are shown in Figure 5.

**Figure 6:**
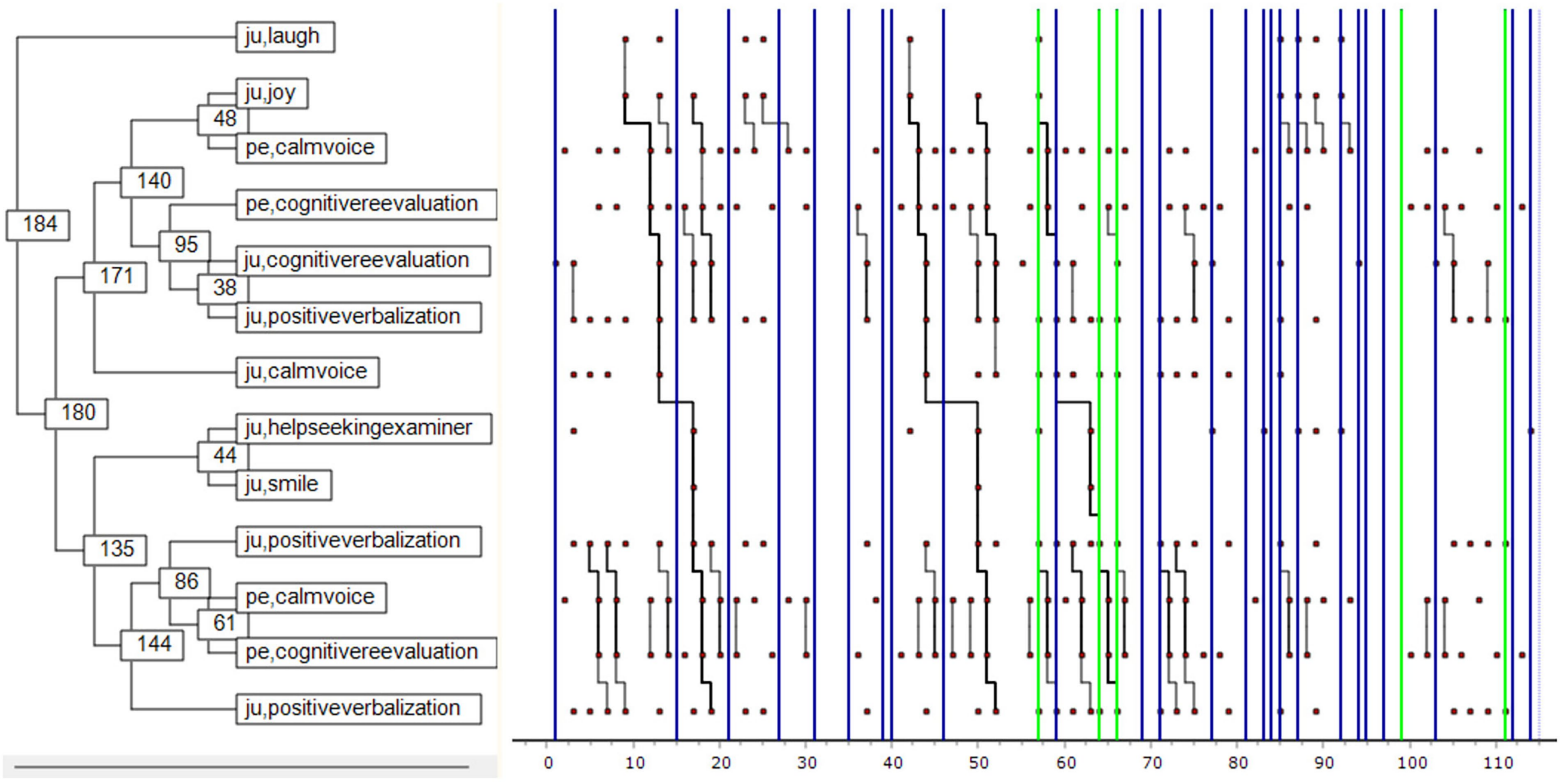
Julia-Father interaction pattern. ju : Julia, pe : father. The X-axis shows the number of behavioral units occurring within the selected 2-min interaction (114 acts overall). Green lines highlight the disengagement behaviors and blue lines are related to self-excitement attitudes. The numbers appearing in the left side of the figure are related to an identification number of each node and is not related with the appearing occurrence of each behavior.

**Figure 7:**
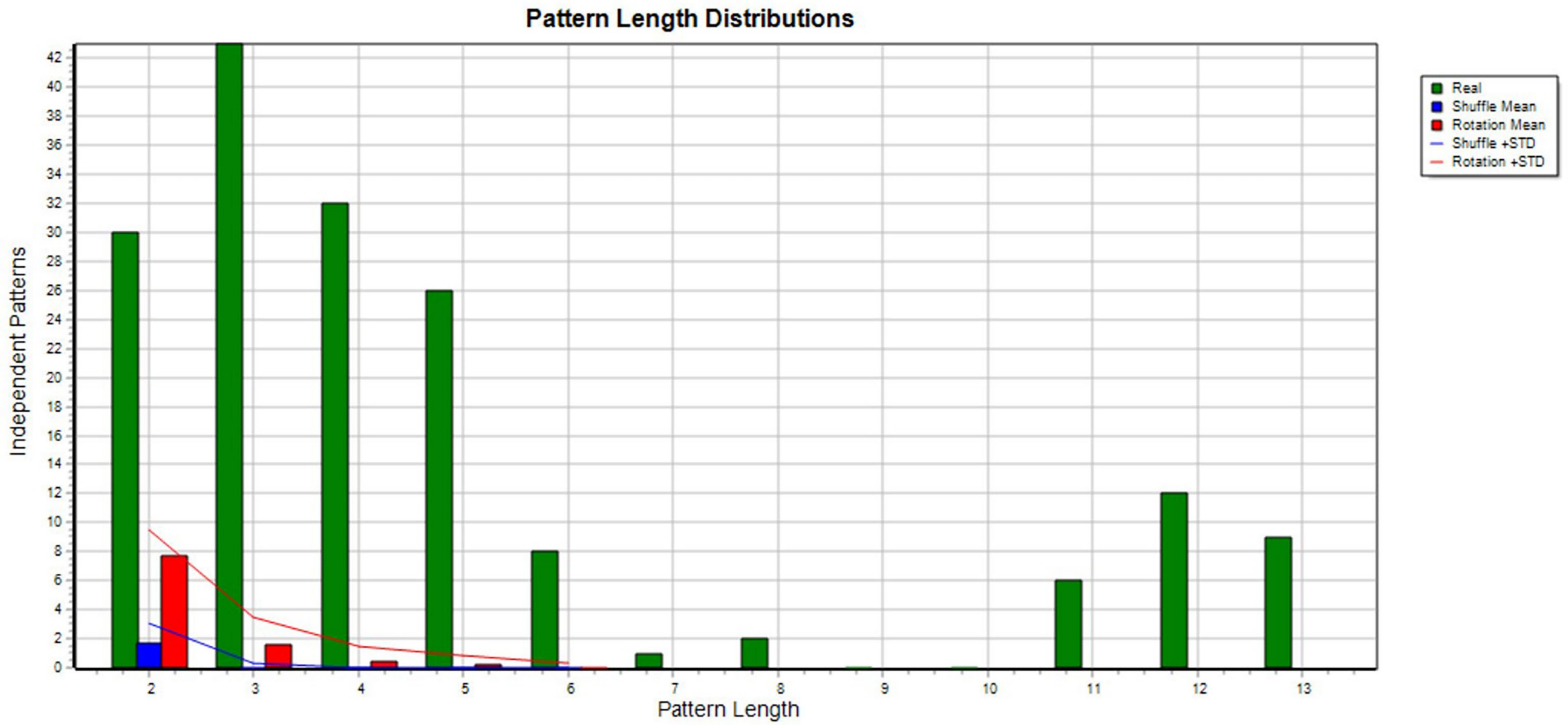
Monte Carlo test results. Shows the results of T-shuffling (blue) and T-rotation (red) for TPA of Julia and her father interactions where 169 patterns were found. The difference between real and randomized data is great and no pattern are found in the randomized data when pattern length increase.

Figure 6 shows the patterns of interaction between Julia and her father. Together they performed 114 acts. It indicates how Julia’s laughter triggers her father to intervene. He speaks calmly, using cognitive reappraisal, stimulating the same process in Julia, who verbalizes positively and elaborates. These behaviors prompt a third-party support-seeking intervention by Julia, who smiles. There follows a repetition of the first pattern, Julia using mental elaboration in a calm and positive exchange with her father. The blue lines indicate Julia’s regular self-excitations. Disengagement occurs but is relatively infrequent, the interaction between Julia and her father taking the form of a coupling of attitudes that includes many mirror reactions. Julia’s need for support within the relationship is apparent in her solicitation of a third party during the episode. We also note the absence of engagement with an object. The Monte Carlo results are shown in Figure 7.

The T-pattern chosen to explore Julia’s interactions with her caregivers did not show the disengagement and self-excitement strategies in the pattern itself. Because those behaviors appeared to occur frequently within the interactions of Julia and her caregivers, they were selected as a marker in the figures presented in the results.

## Discussion

This analysis of interactive patterns between Julia and her adult carers indicates a wide disparity in relational modalities. While communication is based on verbal exchange and mental elaboration between Julia and her foster carer, there is a near absence of exchange between the child and her mother. The relationship between Julia and her father on the other hand is organized around a reactionary symmetry, within which Julia chooses to seek support from a third party. Focused on the various objects available in the foster carer’s home, Julia and her foster carer show real cooperation. Games are jointly developed between them, creating a bilateral relationship with a strong element of collaboration; that it is supported by cognitive reappraisals and regular verbal exchange, positively reinforces the interaction. The emotional atmosphere remains positive, while the attention that the foster carer brings, encourages Julia to develop language ERS within a secure relationship (Labella et al., 2020; Simon-Herrera et al., 2022), an observation that the analysis confirms.

Between Julia and her mother, however, there is an isolating gulf. Julia seems to want to animate the mother figure at all costs, whereas the mother locks herself into a pathological passivity. The relational and emotional detachment manifested by the mother induces violent reactions from Julia who adopts a seriously disorganized attitude. She experiences forms of micro-abandonment during her mother’s visits, while her impulsive behavior – her numerous self-excitations and disengagements – shows the extent of disorganization in her psychic state, a condition that is visibly neither understood nor supported by her mother. These elements support the hypothesis that there is a loop of negativity between the child and her mother – a traumatic repetition from which it is difficult to escape (Vasileva and Petermann, 2017). Self-excitation, although non-violent, may also be interpreted as intense self-aggressive behavior in which Julia relinquishes control over her body and cognitions. The number of engagements with object are up to six while Julia is interacting with her mother, showing a great disorganization of her behavior in a 2-min sequence.

The relationship between Julia and her father could be related to what Bion (1961) called “*coupling*” in his work on small groups. There is a significant similarity and correspondence between the attitudes of Julia and her father, yet the girl feels the need to ask a third party for support – the only relational modality where this occurs. Disengagement remains very present, suggesting that it is difficult for Julia to sustain a stable and prolonged interaction with her father. Thus, despite what seems tacit support between them, their relationship appears organized around attitudes of approach-retreat. The lack of investment in objects indicates that their interaction is focused on themselves and thus on their bodies and speech. Regarding Julia’s ERS, it appears that she presents a disorganized attachment style with multiple aggressive behaviors and a tendency to feel some distress while interacting with her parents.

The analysis of the results shows how Julia’s ER tends to develop differently according to the adult with whom she interacts, as previous case studies have suggested (Simon-Herrera et al., 2022). It is complicated for a foster child such as Julia to develop ERS within multiple parenting systems. It is important to consider as a limitation the fact that a single-case study is presented and the results cannot be globalized. However, the methodology detailed here offered the opportunity to analyze in-depth interaction processes in foster care. The microanalysis carried out with the THEME© software clearly highlights these mechanisms and their disparities according to the type of adult-child relationship, making it an invaluable tool in the search for relational patterns in clinical psychology. These patterns must be understood as interactive loops that are reproduced throughout the exchange (Simon-Herrera et al., 2022).

While disengagement and self-excitement behaviors seemed to be particularly present in the interaction between Julia and her caregivers, no specific pattern showed the process explaining the use of those specific strategies. It can be hypothesized that those behaviors are relatively invasive, especially in mother-daughter interaction, then replicating in other types of relationships. Disengagement behavior is a sign of difficulty in a relationship and indicative of the problem posed by having to maintain contact between a parent and a child at risk, as in the case of Julia and her mother. It questions the whole mother-daughter relationship support system. Since any intervention must have a beginning and an end, the principal difficulty is nonetheless delimiting an objective. In Julia’s case, the highly pathological modalities of the relationship with her mother and the dubious interactions with her father, make it almost impossible to envisage withdrawing the relationship support system. Parent–child visits provoke a form of traumatic reviviscence that impacts on emotional co-regulation (Wekerle et al., 2006): an intervention in the relationship seems inevitable if the child is not to be left in a state of traumatic collapse at each visit. Thus, video feedback can be considered a therapeutic tool that may help an adult to identify, understand and respond to both a child’s, and his or her own, emotional states (Simon-Herrera and Duriez, 2021).

## Data availability statement

The original contributions presented in the study are included in the article/supplementary material, further inquiries can be directed to the corresponding author.

## Ethics statement

Ethical review and approval was not required for the study on human participants in accordance with the local legislation and institutional requirements. Written informed consent to participate in this study was provided by the patient/participants’ or patient/participants legal guardian/next of kin.

## Author contributions

All authors listed have made a substantial, direct, and intellectual contribution to the work and approved it for publication.

## Funding

The present open access publication is funded by the Ecole de Psychologues Praticiens.

## Conflict of interest

The authors declare that the research was conducted in the absence of any commercial or financial relationships that could be construed as a potential conflict of interest.

## Publisher’s note

All claims expressed in this article are solely those of the authors and do not necessarily represent those of their affiliated organizations, or those of the publisher, the editors and the reviewers. Any product that may be evaluated in this article, or claim that may be made by its manufacturer, is not guaranteed or endorsed by the publisher.
